# Relationship Between ABO Blood Groups and Mental Disorders

**DOI:** 10.2147/JBM.S470340

**Published:** 2025-01-03

**Authors:** Vladeta Ajdacic-Gross, Ana Buadze, En-Young N Wagner, Mario Müller, Erich Seifritz, Setareh Ranjbar, Jennifer Glaus, Enrique Castelao, Marie-Pierre F Strippoli, Caroline L Vandeleur, Martin Preisig, Roland von Känel

**Affiliations:** 1 Department of Adult Psychiatry and Psychotherapy, Psychiatric University Clinic Zurich and University of Zurich, Zurich, Switzerland; 2 Department of Consultation-Liaison Psychiatry and Psychosomatic Medicine, University Hospital Zurich and University of Zurich, Zurich, Switzerland; 3 Department of Psychiatry, Psychiatric Epidemiology and Psychopathology Research Center, Lausanne University Hospital and University of Lausanne, Prilly, Switzerland; 4 Department of Psychiatry, Service of Child and Adolescent Psychiatry, Lausanne University Hospital and University of Lausanne, Lausanne, Switzerland

**Keywords:** ABO blood groups, neurodevelopmental disorders, substance use disorders, familial aggregation

## Abstract

Previous research has unveiled an intriguing positive association between the AB blood group and mental disorders in general. In this study, we compared ABO blood groups with five major groups of mental disorders to attain a higher level of specificity. The analyses were conducted using data from the CoLaus|PsyCoLaus study (N=5111). They revealed that the AB blood group exhibited a positive association with both neurodevelopmental disorders (RR 2.29, CI 1.38–3.82) and substance use disorders (RR 2.25, CI 1.38–3.65) after adjusting for sex and childhood adversities. These associations could be replicated with respect to the familial aggregation of neurodevelopmental and substance use disorders. Large databases are needed to achieve more detailed results related to specific disorders.

## Plain Language Summary

Prior research has shown an interesting link between the AB blood group and mental disorders. In our study, we used data from a large Swiss population-based epidemiological cohort study to learn more about this connection. We examined the relationship between ABO blood groups and five major groups of mental disorders: neurodevelopmental, early onset anxiety, later onset anxiety, mood, and substance use disorders. Our analyses revealed associations between the AB blood group and neurodevelopmental and substance use disorders. The findings point to an unknown immunological mechanism that may play a role in the development of neurodevelopmental and substance use disorders.

## Introduction

ABO blood groups are characterized by A- and B-antigens on red blood cells and corresponding antibodies (anti-A, anti-B), which develop early in life through interactions with environmental factors such as nutrition or pathogens.^1^ Research on associations between ABO blood groups and physiological or clinical conditions has a long tradition^2^ and has received renewed due to the availability of large research databases involving genotype-related information such as the UK Biobank.^3^,^4^ However, in psychiatric research, the results have been inconsistent. In a recent analysis, Pisk et al^5^ found a positive association between the AB blood group and mental disorders (overall data) with an odds ratio of about 3. More detailed results could not be obtained due to a relatively small sample and because the AB blood group is the smallest group in Caucasian populations (<5%). In the present study, we went a step further and examined associations between ABO blood groups and five major groups of mental disorders, including neurodevelopmental, early / later onset anxiety, mood, and substance use disorders as introduced in previous studies.^6^ This intermediate-level subgrouping aimed to ensure adequate subsample sizes from the present data. Additionally, it aligns with critical developmental stages: early brain development (neurodevelopmental and early-onset anxiety disorders) and brain maturation (later-onset anxiety, mood, and substance use disorders). In addition to information on the study participants themselves, we included disorder specific information reported on their relatives, thus representing the familial aggregation of mental disorders. The analyses were based on data from a large Swiss population-based epidemiological cohort study.

## Materials and Methods

CoLaus|PsyCoLaus^7^,^8^ is a prospective cohort study that started in 2003, designed to investigate cardiovascular risk factors (CoLaus part) and mental disorders (PsyCoLaus part) in the community and to determine their associations. The initial sample (n=6734) was randomly selected among 35- to 75-year-old residents from the city of Lausanne (Switzerland) according to the civil register. After the first physical and psychiatric assessment, which took place between 2003 and 2008, the cohort was followed-up approximately 5 years (Follow-up 1, FU1) and 10 years (Follow-up 2, FU2) later. The PsyCoLaus subsample with complete baseline data totaled n=5111.

Information on ABO blood groups was based on self-reporting by the CoLaus|PsyCoLaus participants and was available from 3039 (59.5%) participants. Familial aggregation information was assessed by the French version of the semi-structured Family History–Research Diagnostic Criteria (FH-RDC) interview.^9^ The FH-RDC information was assessed only in a sub-sample of CoLaus|PsyCoLaus participants (n=3378 (66.1%)).

The French version^10^ of the semi-structured Diagnostic Interview for Genetic Studies (DIGS)^11^ was used to assess diagnostic information on mental disorders. The assessments covered a broad range of the Diagnostic and Statistical Manual of Mental Disorders IV (DSM-IV) Axis I criteria, as well as the course and chronology of comorbid features. Trained psychologists conducted the interviews, while diagnoses were based on algorithms, similar to those used in other large population-based surveys.^12–15^

In this study, the disorders were categorized following the approach used in previous analyses:^6^ (1) neurodevelopmental, including disruptive behavior disorders typically starting during childhood: tics / Tourette syndrome, attention-deficit / hyperactivity disorder, conduct disorder, oppositional defiant disorder; (2) early onset anxiety disorders typically starting during childhood: separation anxiety disorder, early onset generalized anxiety disorder, animal phobias, social phobia; (3) later onset anxiety disorders typically starting after adolescence: generalized anxiety disorder, panic, agoraphobia, specific phobias (excl. animal phobias); (4) mood disorders: major depressive disorder, dysthymia, bipolar disorders; and (5) substance use disorders: alcohol, cannabis, other illicit drug abuse/dependence. These major groups of mental disorders are not mutually exclusive. The mental disorders assessed with the FH-RDC were grouped in the same way. Given the sample size of the CoLaus|PsyCoLaus study, subgrouping based on a consistent intermediate differentiation level yielded adequate subsample sizes for further analyses. Disorders not covered by this subgrouping approach (eg, obsessive-compulsive disorder, disorders of the schizophrenia spectrum, eating disorders) had subsample sizes that were too small in CoLaus|PsyCoLaus to be included in the analyses.

The statistical analysis relied on basic procedures. Descriptive statistics were provided in terms of frequencies and proportions of the main groups of mental disorders among participants and their relatives. They were compared among the blood groups using the χ^2^ test for contingency analysis. Multinomial logistic regression analysis was used to check the preliminary results by adjusting for sex, childhood adversities and familial aggregation. Childhood adversities were introduced as a sum variable comprising interparental violence, fear from severe punishment, running away from home, death of mother, death of father, foster placement, traumatic experiences before the age of 10.

The authors assert that all procedures contributing to this work comply with the ethical standards of the relevant national and institutional committees on human experimentation and with the Helsinki Declaration of 1975, as revised in 2008. All procedures involving human subjects/patients were approved by the University of Lausanne’s Institutional Ethics Committee.^7^ All participants received a detailed description of the goals, procedures and funding of the study and signed a written informed consent form.

## Results

The frequencies and proportions of the main groups of mental disorders by blood group in PsyCoLaus participants are displayed in Table 1. Three mental disorder groups showed remarkable outcomes among PsyCoLaus participants. In neurodevelopmental and in substance use disorders, the AB blood group was overrepresented compared to other blood groups. This was also consistently observable across specific neurodevelopmental disorders (data not shown). In addition, the AB blood group stood out in substance use disorders, whereas the B blood group had the highest proportions in later onset anxiety disorders.

**Table 1:** Frequencies of Mental Disorders by ABO Blood Group

	N ^1^	A	AB	B	O	χ^2^	d.f.	p-value ^2^
		N (%)	N (%)	N (%)	N (%)			
*Participants*								
Neurodevelopmental disorders	269	111 (8.6)	22 (17.1)	26 (8.2)	110 (8.5)	11.28	3	0.010
Early onset anxiety disorders	718	321 (24.8)	29 (22.8)	71 (22.5)	297 (22.8)	1.82	3	0.611
Later onset anxiety disorders	405	186 (14.4)	11 (8.5)	53 (16.8)	155 (11.9)	9.32	3	0.025
Mood disorders	1378	577 (44.6)	62 (48.1)	146 (46.2)	593 (45.6)	0.78	3	0.855
Substance use disorders	348	152 (11.8)	27 (20.9)	35 (11.1)	134 (10.3)	13.29	3	0.004
*Relatives*								
Neurodevelopmental disorders	239	111 (12.4)	20 (20.8)	20 (10.2)	88 (10.3)	10.14	3	0.017
Early onset anxiety disorders	726	326 (36.4)	42 (43.8)	65 (33.2)	293 (34.4)	4.10	3	0.251
Later onset anxiety disorders	247	114 (12.7)	10 (10.4)	22 (11.2)	101 (11.9)	0.79	3	0.853
Mood disorders	899	396 (44.2)	52 (54.2)	67 (34.2)	384 (45.1)	12.10	3	0.007
Substance use disorders	215	111 (12.4)	12 (12.5)	17 (8.7)	75 (8.8)	7.13	3	0.068

**Notes**: ^1^ the groups are not mutually exclusive. ^2^ p-values of contingency analysis (χ^2^ test): disorder group (y/n) vs ABO blood group.

**Abbreviations**: d.f., degrees of freedom.

In multinomial logistic regression analyses the results did not substantially change despite adjustment for sex and childhood adversities (tables not shown). The relative risk (95% confidence intervals in parentheses) for the AB blood group in neurodevelopmental disorders was 2.29 (1.38–3.82) with blood group O as the reference group. After additionally including familial aggregation (reduced sample), the relative risk was 2.61 (1.49–4.56). The respective relative risks for substance use disorders were 2.25 (1.38–3.65) after basic adjustment (ie, sex, childhood adversities, neurodevelopmental disorders and early onset anxiety disorders) and 1.94 (1.12–3.35) after including familial aggregation (reduced sample).

Frequencies and proportions of the main groups of mental disorders by blood group for relatives are shown in the lower part of Table 1. Consistent patterns as in the CoLaus|PsyCoLaus participants emerged regarding neurodevelopmental and substance use disorders.

## Discussion

This study examined associations between ABO blood groups and five major groups of mental disorders. Previous research had highlighted an association between the AB group and mental disorders.^5^ Here, the analyses detailed that this association is further emerging due to neurodevelopmental and substance use disorders. This specific vulnerability of persons with the AB blood group converges with the finding that the AB group is linked to an increased risk of cognitive impairment.^16^

Blood groups such as the ABO system are inherited from parents. Accordingly, familial aggregation of neurodevelopmental and mental disorders expectably shows partly redundant associations with ABO blood groups. In this analysis, this was demonstrated for the AB group in neurodevelopmental and less clearly in substance use disorders, whereas the replication failed regarding other associations.

A concise interpretation of the findings is not immediately clear. Interestingly, the development of the ABO blood groups is linked to the development of the gastrointestinal microbiota early in life.^1^ In particular, Gram-negative bacteria are believed to stimulate the development of anti-A and anti-B antibodies. The AB blood group is characterized by the lack of the anti-A and anti-B antibodies altogether, thus possibly lacking potentially beneficial immunological stimulation. A similar reasoning was suggested by Flegr et al^17^ who found that Rhesus negative persons encounter more somatic and mental problems than Rhesus positive subjects.

In sum, the findings point to a specific immunological mechanism related to the AB blood group and contributing to the risk for neurodevelopmental and substance use disorders. Similarly, comparable associations might be expected with other disorders and diseases that are linked to insufficient immunological stimulation early in life. The theoretical foundation for this is provided by the hygiene (or old friends) hypothesis,^18^,^19^ which posits that lack of immunological stimulation increases the risk for conditions such as atopic diseases.^20^ Notably, allergic diseases and neurodevelopmental disorders are also associated with each other.^21^,^22^ The challenge of future research on the AB blood group, using very large databases, will be to disentangle the associations with specific neurodevelopmental and schizophrenia spectrum disorders.

## Limitations

The results of this study should be interpreted in the light of notable limitations. The sample size of this study is greater than most other studies on the associations between ABO blood groups and mental disorders; however, it is still underpowered in view of cross-sections between small ABO blood groups (such as the AB group) and less frequent disorders. Therefore, the current analyses were based on five groups of mental disorders.

The information on ABO blood groups and mental disorders in CoLaus|PsyCoLaus relies on self-reporting. Due to recall bias, this study underestimates the prevalence of mental disorders and is skewed towards more burdening symptom configurations. However, there is no apparent systematic bias interfering with the associations between ABO blood groups and mental disorders.

## Data Availability

The data are not publicly available due to restrictions provided in the written consent form signed by the study participants at the beginning of the study (2003-2006).
